# In Situ Endothelial SARS-CoV-2 Presence and PROS1 Plasma Levels Alteration in SARS-CoV-2-Associated Coagulopathies

**DOI:** 10.3390/life14020237

**Published:** 2024-02-08

**Authors:** Marcello Baroni, Silvia Beltrami, Giovanna Schiuma, Paolo Ferraresi, Sabrina Rizzo, Angelina Passaro, Juana Maria Sanz Molina, Roberta Rizzo, Dario Di Luca, Daria Bortolotti

**Affiliations:** 1 Department of Life Sciences and Biotechnology (SVEB), University of Ferrara, 44121 Ferrara, Italy; marcello.baroni@unife.it (M.B.); paolo.ferraresi@unife.it (P.F.); 2 Department of Chemical, Pharmaceutical and Agricultural Sciences, University of Ferrara, 44121 Ferrara, Italy; silvia.beltrami@unife.it (S.B.); giovanna.schiuma@unife.it (G.S.); sabrina.rizzo@unife.it (S.R.); juana.sanz@unife.it (J.M.S.M.); brtdra@unife.it (D.B.); 3 Department of Translational Medicine, University of Ferrara, 44121 Ferrara, Italy; angelina.passaro@unife.it; 4 Department of Medical Sciences, University of Ferrara, 44121 Ferrara, Italy; ddl@unife.it

**Keywords:** coagulopathy, PROS1, PLpro, SARS-CoV-2, COVID-19

## Abstract

Background: Coagulation decompensation is one of the complications most frequently encountered in COVID-19 patients with a poor prognosis or long-COVID syndrome, possibly due to the persistence of SARS-CoV-2 infection in the cardiovascular system. To date, the mechanism underlying the alteration of the coagulation cascade in COVID-19 patients remains misunderstood and the anticoagulant protein S (PROS1) has been described as a potential risk factor for complications related to COVID-19, due to PLpro SARS-CoV-2 enzyme proteolysis. Methods: Biopsies and blood samples were collected from SARS-CoV-2 positive and negative swab test subjects with coagulopathies (peripheral arterial thrombosis), and SARS-CoV-2 presence, ACE2 and CD147 expression, and plasmatic levels of PROS1 were evaluated. Results: We reported a significant decrease of plasmatic PROS1 in the coagulopathic SARS-CoV-2 swab positive cohort, in association with SARS-CoV-2 in situ infection and CD147 peculiar expression. These data suggested that SARS-CoV-2 associated thrombotic/ischemic events might involve PROS1 cleavage by viral PLpro directly in the site of infection, leading to the loss of its anticoagulant function. Conclusions: Based on this evidence, the identification of predisposing factors, such as CD147 increased expression, and the use of PLpro inhibitors to preserve PROS1 function, might be useful for COVID-19 coagulopathies management.

## 1. Introduction

The broad tropism of Severe Acute Respiratory Syndrome virus 2 (SARS-CoV-2) contributes to the manifestation of various diseases associated with this infection. This is attributed to the widespread presence of specific receptors for SARS-CoV-2 on human cells [1,2]. SARS-CoV-2 enters host cells by utilizing the angiotensin-converting enzyme 2 (ACE2), which is primarily expressed in lung cells, cardiac myocytes, vascular endothelium, kidney, heart, gastrointestinal tract, pancreas, and testicles [3]. Apart from ACE2, the CD147 receptor has also been identified as a potential receptor for the virus [4]. CD147, also known as extracellular matrix metalloproteinase inducer (EMMPRIN), is a member of the immunoglobulin superfamily and is expressed at varying levels in diverse cell types, including hematopoietic, epithelial, endothelial cells (ECs), and leukocytes [4].

Despite the widespread presence of SARS-CoV-2 receptors throughout the body, the virus primarily targets the upper and/or lower respiratory tract, resulting in fever, dry cough, fatigue, dyspnea, diarrhea, headache, and myalgia [5]. Some individuals recovering from COVID-19 may develop Long COVID-19 syndrome (LCS), leading to long-term complications [6,7] in various body systems beyond the respiratory tract [8,9]. LCS can impact respiratory, cardiovascular, renal, neurological, hematological, and digestive sites, but it remains poorly understood. Recent findings indicate the presence of SARS-CoV-2 in the gastrointestinal tract of individuals previously positive for the virus, particularly those hospitalized for abdominal thrombosis. This long-term manifestation of COVID-19 is associated with unique CD147 and VEGF expression, potentially contributing to hemostatic and vascular alterations [10,11]. Complications such as coagulopathies (thrombosis, disseminated intravascular coagulation), immune and inflammatory activation play a crucial role in the rapid deterioration of the patient’s clinical condition [12], representing the second leading cause of death during SARS-CoV-2 infection [13,14].

At the basis of the manifestation of thrombotic events associated with COVID-19 disease there is an important imbalance of the hemostatic system, also due to the ability of SARS-CoV-2 to infect both vascular endothelial cells and platelets [15]. Although SARS-CoV-2 infection in endothelial cells is non-productive [16], it may induce cell modifications contributing to adverse cardiovascular events typical of COVID-19. In response to direct or indirect viral exposure, endothelial cells produce a pro-inflammatory response [17,18], that provokes an increased production of thrombin, blocking fibrinolysis thereby determining a hypercoagulability condition [18]. For this reason, COVID-19 hospitalized patients often showed alterations of several coagulation parameters, such as activated partial thromboplastin time (aPTT), prothrombin time (PT), fibrinogen, platelet count, fibrin degradation products (FDP), D-dimer, von Willebrand factor, factor VIII, factor V, factor II, tissue factor, antithrombin, thrombomodulin and protein S [19]. Moreover, COVID-19 patients present hyperactivated ACE2-positive [20] and CD147-positive [21] platelets, which supports the notion of SARS-CoV-2 directly participating in the observed thrombus formation and inflammation in COVID-19 subjects.

Recently, protein S1 (PROS1) has been described to be involved in the occurrence of coagulopathies associated with COVID-19 [22]. PROS1 is a vitamin K-dependent plasma glycoprotein that is primarily synthetized by the endothelium [23] and megakaryocytes [24] which plays a key role in natural anticoagulant processes. It functions as a cofactor for activated protein C (APC), enhancing APC’s ability to inhibit blood clot formation by inactivating coagulation factors Va and VIIIa. PROS1 acts as a key regulator in maintaining a delicate balance between procoagulant and anticoagulant forces, preventing excessive blood clotting and thrombosis. Alterations in PROS1, whether due to genetic mutations or acquired deficiencies, can lead to an increased risk of venous thrombosis. When PROS1 is compromised, the anticoagulant activity of APC is impaired, allowing unchecked coagulation processes that may result in abnormal blood clot formation and a higher risk of thromboembolism development [25].

While the specific role of PROS1 in SARS-CoV-2 coagulopathies is an area of ongoing research, its potential involvement in the context of COVID-19-associated clotting disorders is noteworthy. SARS-CoV-2 infection has been linked to a heightened risk of coagulopathies, including thrombosis and disseminated intravascular coagulation (DIC). PROS1, as a key regulator of anticoagulant processes, may play a role in mitigating these coagulation abnormalities.

The impact of SARS-CoV-2 on PROS1 activity has been substantiated through the observation of reduced activity in 65% of COVID-19 patients [26]. This phenomenon is attributed to the papain-like protease (PLpro) of SARS-CoV-2, which has been identified as a potential contributor to thrombotic hypercoagulation and deregulation. PLpro modifies PROS1 antithrombotic and immunomodulatory properties by proteolytical cleavage [24,27]. The validity of this hypothesis was further confirmed through in vitro experiments, which demonstrated the cleavage of PROS1 occurring in proximity to viral replication complexes [26,28]. Therefore, the modification of PROS1 expression by PLpro within platelets could disturb thrombin formation and activate platelets, potentially contributing to the disruption of various platelet functions observed in COVID-19 patients, playing a role in the formation of thrombi [29].

As of now, there is no available information on the potential direct link between in situ vascular infection by SARS-CoV-2 and the impairment of PROS1 in COVID-19 patients with coagulopathies. Given the increasing focus on cardiovascular long-term effects of COVID-19, this study seeks to elucidate the potential connection between alterations in PROS1 plasma levels and the concomitant presence of the virus within cardiovascular tissues, following past or ongoing SARS-CoV-2 infection. The goal is to identify novel clinical biomarkers that can aid in the management of coagulopathies associated with COVID-19.

## 2. Materials and Methods

### 2.1. Patients and Samples Collection

The study was conducted on 18 patients affected by coagulopathies (peripheral arterial thrombosis) enrolled from May to December 2020 at the Internal Medicine Unit of the Sant’Anna University Hospital in Ferrara. All the patients showed arterial coagulation, 56% were subjected to thromboendarterectomy and 17% were subjected to amputation. All the subjects were free from any medication before the sample collection and patients with concomitant comorbidities, such as BCPO, diabetes or autoimmune diseases, were excluded. In particular, two cohorts were identified: 7 patients had at least one SARS-CoV-2 positive oropharyngeal swab within 6 months of the coagulopathy event, experiencing mild symptoms, and 11 patients had never reported a SARS-CoV-2 positive oropharyngeal swab. No subjects were vaccinated for SARS-CoV-2, or present previous comorbidities or treatments (e.g., anticoagulants, antiplatelet agents). The study was approved by our hospital’s ethics committee (Number: 540/2020/Oss/AOUFe—20 May 2020). All the data were anonymized and no connection with the patient’s identity was possible. For each patient we collected arterial (endothelial tissue) and thrombotic (endovascular thrombotic material) material. Tissue samples were used for total RNA extraction with TRIZOL or fixed in formalin and embedded in paraffin and processed to obtain 4 µm thick sections for histological evaluation and immunohistochemistry. Plasma samples were collected from all the patients and used for ELISA assay.

### 2.2. Histology and Immunohistochemistry

Immunohistochemical (IHC) analysis was performed on the collected samples for detection of SARS-CoV-2 NP (NB100-56576, Novus Biologicals, Centennial, CO, USA, Centennial, 1:250 dilution), CD147 (clone MEM-M6/1, dilution 1:100, Novus Biologicals) and ACE2 (clone EPR4435-2, 1:250 dilution, Abcam, Cambridge, UK), using the Ultratek kit (Histoline, Milan, Italy), as previously described [11]. After immunohistochemical staining, tissue images were analyzed by QuPath software v2.3 and scored based on the intensity and number of positively stained cells/mm^2^ (H-score).

### 2.3. Real-Time PCR

The presence of SARS-CoV-2 genome, targeting the viral spike RBD (Receptor Binding Domain), ACE2 and CD147 in the histological tissues of thrombi and venous/arterial samples was detected by RT-qPCR. cDNA has been synthesized via reverse transcription using the High Capacity kit (ThermoFisher, Scientific, Waltham, MA, USA), from the extracted RNA and gene expression detected by amplification with QuantStudio3 (Thermo Fisher Scientific, Waltham, MA, USA), using PowerUp SYBR Green Master Mix. SARS-CoV-2 RBD domain were amplified using primer forward (5′-CAA TGG TTT AAC AGT CAC AGG-3′) and reverse (5′-CTC AAG TGT CTG TGG ATC ACG-3′); ACE2, CD147 and GAPDH, as housekeeping gene, were detected using PrimeTime primer sets (ACE2: Hs.PT.58.27645939; CD147: Hs.PT.56a.39293590.g; GAPDH: Hs.PT.39.22214836), as previously described [11,30].

### 2.4. PROS1 ELISA Assay

Polyclonal sheep anti-human protein S (PS) antibody (4 mg/L, H.T.I., Huntington, VT, USA) was coated overnight at 4 °C to microtiter plate (Nunc, MaxiSorp^®^, ThermoFisher, Scientific, Waltham, MA, USA) in a 50 mM Na_2_CO_3_/NaHCO_3_, 5 mM CaCl_2_, pH 9.0 buffer, and incubated for 75′ at room temperature (r.t.) with increasing concentrations of PS (0–400 μg/L), from human pooled normal plasma. Bound PS, from 1/2000 diluted plasma samples, was detected with 60′ of incubation at r.t. with polyclonal rabbit anti-PS antibody (4 mg/L, Dako, Glostrup, Denmark) and with a polyclonal goat peroxidase-conjugated antibody (0.6 mg/L, Dako), both incubated at r.t for 60′. A 5 mg tablet OPD (Sigma-Merck, Darmstadt, Germany), dissolved in 12 mL of 50 mM citrate–phosphate buffer pH 5.0 and 7 μL of 30% H_2_O_2_, was added as substrate for peroxidase. After 5′, the reaction was quenched with the addition of 2.5 M H_2_SO_4_ and the color produced was quantified using a SpectraFluor Plus microplate reader (Tecan, Salzburg, Austria), measuring the absorbance at 492 nm. A mix of 50 mM Tris, 150 mM NaCl, 5 mM CaCl_2_, pH 7.4 was used to prepare the blocking, or the sample diluent, or the washer buffers by the addition of 5% albumin from bovine serum (BSA, Sigma-Merck, St. Louis, MO, USA), or 0.2% BSA, or 0.1% Tween 20 (Sigma-Merck), respectively. All buffers were 0.2 μm filtered. The assay showed very high sensitivity, it allows the recognition of PROS1 in plasma diluted up to 16,000 times (Supplementary Figure S1a) and the specificity is given to the assay by the double recognition based on two polyclonal antibodies (ELISA sandwich) [31]. No cross-reactivity was evident in thousands of samples (media and plasma) evaluated and no differences in PROS1 or PROS1-C4BP complex recognition (Supplementary Figure S1b). The inter-assay coefficient of variability (1.69%) was calculated by 3 independent assays performed with 3 samples, quantified in triplicate.

### 2.5. Statistical Analysis

Biological variables reported as frequency were analyzed by Fisher exact test or Chi square test. Biological variables reported as mean ± SD were compared between study groups by Student’s *t*-test. The statistical analysis was performed by GraphPad Prism v.9 Software.

## 3. Results

### 3.1. Characterization of the Study Population

The patients were subdivided based on reported SARS-CoV-2 positive swabs (Table 1). The SARS-CoV-2 Alpha VOC (variant of concern) was detected in all the samples. As reported in Table 1, the patients with SARS-CoV-2 positive swab showed a higher mean age compared to patients with no SARS-CoV-2 positive swab (*p* < 0.0001; Student’s *t*-test), while no significant differences in gender ratio was observed. Both cohorts underwent thromboendoarterectomy, with only two patients with previous SARS-CoV-2 positive swabs characterized by the need of amputation (Table 1, *p* < 0.0001; Fisher exact test).

### 3.2. SARS-CoV-2 In Situ Presence Correlates with Previous Infection in Coagulopathic COVID-19 Subjects

**Figure 1:**
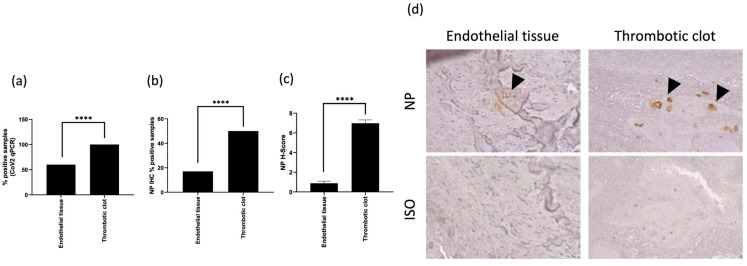
SARS-CoV-2 in situ infection evaluation: (**a**) percentage of positivity for SARS-CoV-2 RBD spike protein in tissues analyzed by Real-Time PCR; (**b**) percentage of positivity for SARS-CoV-2 NP in tissues analyzed by IHC staining and (**c**) corresponding H-Score; and (**d**) representative IHC images for SARS-CoV-2 NP (NP) and isotype control (ISO) in endothelial tissue and thrombotic clot. **** *p* < 0.0001, Chi square test.

RNA samples were obtained from arterial biopsies, which were composed of the endothelial tissues and the thrombotic clots. The samples from subjects with previous SARS-CoV-2 positive swab tests were evaluated for the expression of viral RNA encoding spike protein RBD by RT-qPCR. We found the presence of SARS-CoV-2 RNA encoding spike protein RBD in the 50% of the endothelial tissue and in the totality of the thrombotic clots. (Figure 1a, *p* < 0.0001; Chi square test).

We observed the expression of SARS-CoV-2 NP in the 15% of the endothelial tissues and in the 50% of the thrombotic clots (Figure 1b, 50%; *p* < 0.0001; Chi square test). The H score, accounting for the intensity and the proportion of NP expression, was higher in thrombotic clots than in endothelial tissues (Figure 1c; H-score 6.98 ± 0.34 vs. 0.88 ± 0.21, respectively; *p* < 0.0001 Student’s *t*-test). The biopsies were analyzed for the expression of SARS-CoV-2 NP by IHC (Figure 1d).

The higher positivity rate found by RT-PCR analysis in all the specimens in comparison with IHC staining might be associated with both a higher sensibility of RT-PCR analysis, or with a lower SARS-CoV-2 protein translation.

### 3.3. CD147 Expression Correlates with SARS-CoV-2 In Situ Infection in Coagulopathic COVID-19 Subjects

**Figure 2:**
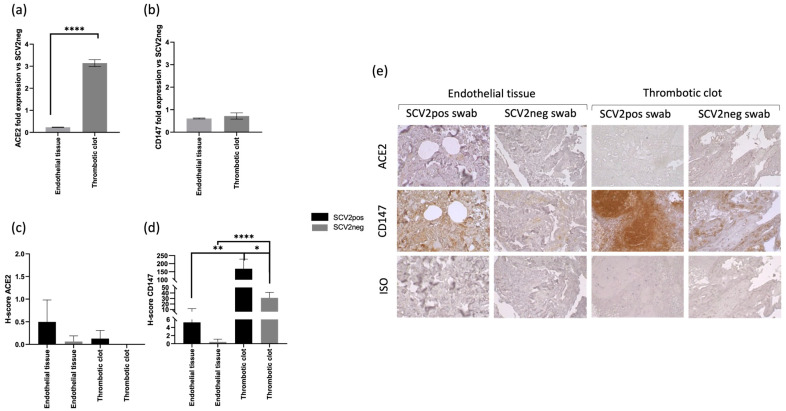
ACE2 (**a**) and CD147 (**b**) fold expression in endothelial tissues and thrombotic clots from SARS-CoV-2 swab positive coagulopathic subjects evaluated by Real Time PCR. The ACE2 and CD147 expression in endothelial tissues and thrombotic clots from SARS-CoV-2 swab negative patients (SCV2neg) were considered as reference levels; (**c**) ACE2 and (**d**) CD147 IHC staining intensity reported as H-Score in endothelial tissues and thrombotic clots from positive (SCV2pos) and negative (SCV2neg) SARS-CoV-2 swab coagulopathic subjects; (**e**) representative IHC staining for ACE2, CD147 and isotype control (ISO) in endothelial tissues and thrombotic clots from positive (SCV2pos) and negative (SCV2neg) SARS-CoV-2 swab coagulopathic subjects. * *p* < 0.05; ** *p* < 0.01; **** *p* < 0.0001, Student’s *t*-test.

The presence of both SARS-CoV-2 RNA and protein expression suggests the ability of the virus to infect the analyzed tissues. Since the viral infection of a host cell depends on the presence of specific receptors, we analyzed the expression of two of the main viral entry receptors, ACE2 and CD147 [4,32], at both the transcriptional and protein level (Figure 2).

The analysis of mRNA levels by ”real time PCR revealed an increased expression of ACE2 mRNA in thrombotic clots of the subjects with previous SARS-CoV-2 positive swab tests in comparison with the subjects with negative SARS-CoV-2 swab tests (Figure 2a, *p* < 0.0001; Student’s *t*-test). Interestingly, the analysis of ACE2 and CD147 protein expression by IHC showed the highest levels in subjects with previous SARS-CoV-2 positive swab tests, in the endothelial tissues for ACE2 and in thrombotic clots for CD147 (Figure 2c–e; *p* < 0.01; Student’s *t*-test).

These data are in agreement with the in situ presence of SARS-CoV-2 RNA and NP protein (Figure 1), suggesting a possible in situ infection, as already described [15].

### 3.4. SARS-CoV-2 In Situ Infection Correlates with Lower Plasma Levels of PROS1

SARS-CoV-2 in situ endothelial infection is known to lead to an abortive infection [16], even affecting the proteome expression of the infected endothelial cells. The main effect seems to be on the inflammatory and angiogenetic processes, accounting for the presence of a basal viral protein expression that might alter cellular functions.

PROS1 has been demonstrated to affect hemostatic regulation, due to its interaction with protein C (APC) and tissue factor pathway inhibitor (TFPI), inducing an antithrombotic/anti-coagulative pathway [24,27]. We evaluated the levels of PROS1 by ELISA in the plasma samples of SARS-CoV-2 positive and negative swab test patients.

**Figure 3:**
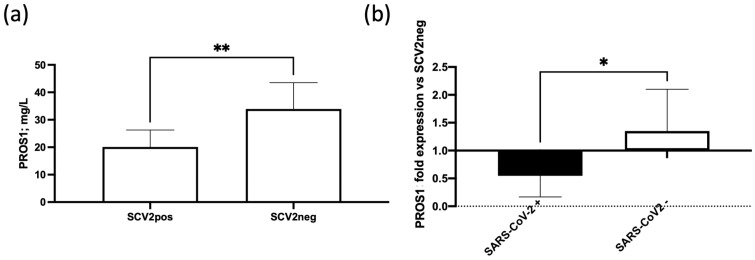
(**a**) Plasma concentration of PROS1 in SARS-CoV-2 positive (SCV2pos) and negative (SCV2neg) swab test patients; (**b**) PROS1 plasma levels in SARS-CoV-2 positive (SCV2pos) swab test patients, subdivided according to the positivity (SARS-CoV-2+) or negativity (SARS-CoV-2-) of the bioptic tissues for SARS-CoV-2 RBD spike RNA, in comparison with PROS1 plasma levels in SARS-CoV-2 positive (SCV2pos) swab test patients’ fold difference considering the presence of SARS-CoV-2 (SARS-CoV-2+) or absence (SARS-CoV-2-) in the genome in the biopsies analyzed by Real Time PCR in comparison with the levels in plasma samples from SARS-CoV-2 negative (SCV2neg) swab test patients. * *p* < 0.05; ** *p* < 0.01, Student’s *t*-test.

PROS1 plasma levels were significantly lower in SARS-CoV-2 positive swab test patients in comparison with SARS-CoV-2 negative swab test patients (Figure 3a *p* < 0.01; Student’s *t*-test). When the SARS-CoV-2 positive swab test patients were subdivided according with the in situ SARS-CoV-2 RBD spike protein RNA positivity or negativity, we observed significantly lower PROS1 plasma levels in patients with SARS-CoV-2 positivity (Figure 3b, *p* < 0.05; Student’s *t*-test).

## 4. Discussion

Despite several evidence reporting SARS-CoV-2 tropism for the cardiovascular system [12,13], little is known about coagulopathies development. Recent findings reported that SARS-CoV-2 gastrointestinal in situ infection was related to abdominal bleeding and ischemia [33], suggesting that previous SARS-CoV-2 infection could develop a secondary effect at the vascular level, exploiting specific tissues as a reservoir of infection. Drawing from this understanding, it is conceivable to propose a chronological association between SARS-CoV-2 infection and the development of coagulopathies. This connection involves the individual susceptibility to viral infection, influenced by the expression of viral receptors and predisposition to coagulopathies, which may include potential mutations or functional alterations in PROS1. Additionally, the direct impact of viral infection on the coagulation cascade, including PLpro cleavage on PROS1, could collectively contribute to determining the risk of coagulopathies in COVID-19. These elements might underlie the cardiovascular events observed in LCS, where individuals, despite exhibiting mild symptoms during the acute infection, may experience the activation of prothrombotic pathways due to the persistent presence of the virus. In this study, we analyzed SARS-CoV-2 positive and negative swab patients, who experienced peripheral arterial thrombosis, to evaluate SARS-CoV-2 in situ presence and its possible role in vascular damage. Both groups of patients underwent thromboendoarterectomy, with SARS-CoV-2-positive swab patients characterized by a higher frequency of amputation. We enrolled patients with no previous comorbidities and treatments to avoid confounding variables. The development of coagulopathy is observed to occur subsequent to the confirmation of a positive swab result in individuals belonging to the group with SARS-CoV-2-positive swabs. This temporal association suggests that the manifestation of coagulopathic events is closely linked to the presence and detection of the SARS-CoV-2 virus. This observation highlights the dynamic nature of the relationship between viral infection and the onset of coagulation disorders, emphasizing the need for vigilant monitoring and timely interventions following a positive diagnosis to address and manage potential coagulopathic complications effectively. The understanding of this temporal sequence is crucial in refining clinical strategies and tailoring therapeutic approaches for individuals diagnosed with COVID-19, ensuring a comprehensive and timely response to mitigate the risk of coagulopathy-related complications. Biopsies from Alpha VOC SARS-CoV-2-positive swab patients were found positive for SARS-CoV-2 in situ presence, reporting both viral genome and NP protein expression mainly in thrombotic clots. The evaluation of the effect of different SARS-CoV-2 VOCs might be of extreme interest in understanding how they impact the infection outcome and persistence at the cardiovascular level.

**Figure 4:**
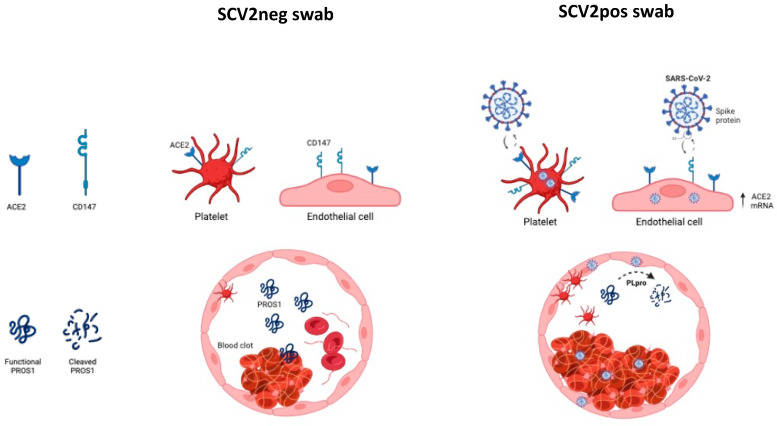
Schematic representation of blood clot formation in no COVID-19 and COVID-19 subjects.

The presence of SARS-CoV-2 in endothelial and thrombotic tissues is supported by an increased expression of both ACE2 and CD147 molecules. Interestingly, we reported a differential tissue expression of these two viral receptors in SARS-CoV-2-positive swab subjects, with a higher amount of ACE2 in the endothelial tissues, while CD147 was more expressed in thrombotic clots. ACE2 is known to be highly expressed on endothelial cells [34], where it represents the main receptor for virus entry [35]. Thus, ACE2 protein higher expression in endothelial tissues from SARS-CoV-2-positive swab patients could support the presence of both SARS-CoV-2 RBD RNA and NP protein at a vascular level. The increased CD147 expression at the thrombotic level might be due to the presence of platelets, that are known to express this receptor [36]. Interestingly, it has been shown that CD147 is able to induce ACE2 surface expression in infected cells [20,37], affecting virus entry into host cells (Figure 4).

The increased CD147 expression in SARS-CoV-2 positive thrombotic clots suggests a critical role in increasing SARS-CoV-2 in situ susceptibility, as already reported for gut viral tropism [11], possibly connected with a higher risk for developing thrombotic events (Figure 4).

This hypothesis is supported also by recent evidence suggesting a role for CD147 in both thrombosis and inflammation, establishing CD147+ platelets as a critical factor in SARS-CoV-2-dependent thrombotic events [36]. Indeed, the thrombotic events associated with COVID-19 are verified to exhibit an atypical state of hyperactivated platelets, linked to the surface expression of CD147 [38]. This reaffirms the involvement of platelets in the inflammatory and hemostatic aspects of the disease. Additionally, there is a proposed significance of the direct interplay between SARS-CoV-2-activated platelets and leukocytes in the formation of blood clots and the onset of a cytokine storm. This supports the consideration of antiplatelet therapies as a potential approach to counteract both thrombotic events and the spread of the infection [39]. These therapies emerge as a promising approach not only to mitigate the thrombotic events associated with COVID-19 but also to impede the spread of the infection. By targeting platelet activation, antiplatelet agents may not only address the hemostatic complications seen in COVID-19 patients, but also disrupt a crucial mechanism implicated in the inflammatory response and viral propagation. The exploration of antiplatelet therapies represents a multifaceted strategy in managing both the vascular complications and the infectious aspects of COVID-19, offering a comprehensive approach to enhance patient outcomes.

As a proof of concept of the possible implication of SARS-CoV-2 infection in coagulopathies, we evaluated the plasmatic levels of the anticoagulant factor PROS1 in correlation to infection status [22], showing lower levels in SARS-CoV-2 positive swab test patients in comparison with SARS-CoV-2 negative swab test patients, in line with previous results [11]. PROS1 is primarily synthesized in the liver, serving as a key site for its production. Additionally, its synthesis occurs locally in other crucial cellular components, such as the endothelium and megakaryocytes, which are the precursors of platelets. This diversified origin of PROS1 underscores its significance in various physiological processes, with contributions from both hepatic and non-hepatic sources, including the endothelial lining and megakaryocytic lineage, thereby emphasizing its multifaceted roles in hemostasis and thrombosis regulation. Furthermore, it has been documented that PROS1 undergoes proteolytic cleavage by the SARS-CoV-2 PLpro viral protease [40]. The observed decrease of PROS1 anticoagulant protein in SARS-CoV-2 positive biopsies strengthens the association between coagulopathies development and SARS-CoV-2 in situ presence, possibly through PROS1 impairment by proteolytic activity, carried out by SARS-CoV-2 PLpro viral protease [40] (Figure 4). The PLpro enzymatic modification of PROS1 raises significant clinical implications. The cleavage of PROS1 by PLpro may disrupt its anticoagulant and immunomodulatory functions, potentially contributing to the hypercoagulable state observed in COVID-19 patients. This molecular interaction highlights a potential mechanism by which SARS-CoV-2 may directly impact the intricate balance of hemostasis, providing valuable insights for understanding the pathophysiology of COVID-19-associated coagulopathies and suggesting new avenues for targeted therapeutic interventions aimed at restoring or mitigating PROS1 functionality. These therapeutic strategies may involve the development of specific inhibitors against the SARS-CoV-2 PLpro protease to prevent its cleavage of PROS1. Additionally, approaches that enhance PROS1 production or stability could be explored to counteract the detrimental effects of its cleavage. Investigating these novel therapeutic avenues holds promise for mitigating the hypercoagulable state associated with COVID-19, ultimately improving patient outcomes, and informing the development of tailored interventions in the evolving landscape of COVID-19 treatment strategies.

This hypothesis necessitates thorough validation through extensive investigations into the molecular mechanisms underlying the observed effects, aiming to offer additional insights into the intricate pathways involved. Specifically, future research endeavors should concentrate on elucidating the impact on the plasmatic levels of PROS1 in the presence of various SARS-CoV-2 VOCs. Focusing on these specific viral variants will allow a more nuanced understanding of how they potentially modulate PROS1 function, shedding light on any variant-specific effects on coagulation and hemostasis. This nuanced exploration is crucial for advancing our comprehension of the interplay between SARS-CoV-2 and PROS1, contributing to the refinement of targeted therapeutic strategies and informing public health measures tailored to the evolving landscape of viral variants and associated clinical manifestations.

Our study is constrained primarily by the limited number of participants included, the single center enrollment and the lack of quantification for the SARS-CoV-2 PLpro viral protease. Additionally, we did not assess inflammatory cytokine plasma levels or conduct coagulative assays. Specifically, a notable absence is the evaluation of plasma PROS1 activity, which could significantly enhance our comprehension of PROS1’s physiological and pathological functionality in individuals with COVID-19. A more extensive enrollment of patients and the inclusion of quantitative measures for viral protease and other relevant factors would contribute to a more robust analysis. Furthermore, incorporating assessments of inflammatory markers and coagulation assays could provide a comprehensive understanding of the complex interplay between the virus and the host’s hemostatic system, offering valuable insights into potential therapeutic interventions and improving the clinical relevance of our findings. The absence of long-term follow-up data in this study poses significant implications for understanding the persistence or resolution of coagulopathies over an extended period in individuals with SARS-CoV-2 infection. The dynamics of coagulation abnormalities in COVID-19 patients can vary over time, and a lack of prolonged observation hinders our ability to comprehensively assess the trajectory and outcomes of these complications. Long-term follow-up data and replication of this study in diverse settings are crucial to unravel the evolving nature of coagulopathies associated with SARS-CoV-2, shedding light on whether these abnormalities persist, resolve, or potentially reoccur after the acute phase of infection.

Nevertheless, the findings are noteworthy as they, for the first time, suggest a direct link between the in situ vascular presence of SARS-CoV-2 and the onset of coagulopathies, proposing an alteration in PROS1 expression as a potential key contributor to SARS-CoV-2-associated coagulation disorders. Identifying predisposing factors, such as the increased expression of CD147 and the reduction in plasmatic PROS1 levels, could be instrumental in proactively determining the risk of coagulopathies associated with SARS-CoV-2 infection and LCS. Monitoring altered plasmatic levels of PROS1 or incorporating its assessment during therapy could aid in identifying COVID-19 subjects at higher risk for PROS1-associated coagulopathies, which can significantly exacerbate the clinical course in COVID-19 patients. Screening for PROS1 mutations could be considered an additional preventive measure against coagulopathic events. Furthermore, recognizing the distinctive heterogeneity in clinical manifestations of COVID-19 is crucial in evaluating the onset of coagulopathic complications. Early assessment of PROS1 levels may contribute to reducing the incidence of COVID-19-associated cardiovascular events, particularly in LCS cases, by facilitating the early identification of the most susceptible patients.

## 5. Conclusions

In this study, we have unveiled, for the first time, a potential association between the in situ presence of SARS-CoV-2 at the cardiovascular level and thrombotic events involving PROS1. A discernible predisposing factor appears to be the distinctive expression patterns of ACE2 and CD147 receptors, with CD147 notably prevalent in thrombotic clots. This heightened presence potentially facilitates viral in situ replication, subsequently intensifying viral PLpro cleavage on PROS1. These findings underscore the pivotal role of SARS-CoV-2 infection in influencing PROS1 plasmatic levels, emerging as a significant risk factor for coagulopathies. Considering this, assessing PROS1 plasmatic levels and CD147 expression emerges as a potent diagnostic and therapeutic tool for COVID-19 patients, providing insights into the risk for SARS-CoV-2-associated coagulopathies. Furthermore, considering these revelations, the exploration of antiplatelet therapies becomes a valuable avenue for countering both thrombotic events and the propagation of infection in COVID-19 patients. To enhance the robustness of these findings, a replication of the data on a broader population is imperative, coupled with a comprehensive long-term follow-up investigation to elucidate the sustained impact of the PLpro viral protease on controlling PROS1 plasmatic levels.

## Figures and Tables

**Table 1:** Demographical and clinical characterization of subjects enrolled in the study.

	SARS-CoV-2 Positive Swab (*N* = 7)	SARS-CoV-2 Negative Swab (*N* = 11)	*p* Values
Gender; N; %	M (5; 71%) F (2; 29%)	M (9; 82%) F (2; 18%)	0.51
Age; mean ± SD	85.0 ± 3.3	70.8 ± 6.7	<0.0001
Thromboendarterectomy	57% (4)	55% (6)	0.65
Amputation	29% (2)	0% (0)	<0.0001

## Data Availability

The data presented in this study are available on request from the corresponding author.

## Supplementary Materials

The following are available online at https://www.mdpi.com/article/10.3390/life14020237/s1, Figure S1: PROS1 ELISA sensitivity and specificity profile. (a) Increasing dilutions of pooled normal plasma (1/31.25—1/16,000, from 800 ng/mL to 1.56 ng/mL of PROS1) in PROS1 deficient plasma, were evaluated by ELISA sandwich, based on polyclonal antibodies. The very high reproducibility intra-assay is visible in the figure and the inter-assay variability is only 1.69%. Each point is the average of 3 measurements; (b) ELISA-PROS1 assay was conducted in pooled normal plasma by addition of C4BP protein (400 ng/mL, gray dashed line) or sample diluent (50 mM Tris, 150 mM NaCl, 5 mM CaCl2, pH 7.4, gray line) and no effect of this addition of this plasmatic physiological interactor of PROS1 was evaluable. This ELISA recognizes free and C4BP-bound PROS1 with identical affinity. Each point is the average of 2 evaluations.
