# Supplementary material for: In Situ Endothelial SARS-CoV-2 Presence and PROS1 Plasma Levels Alteration in SARS-CoV-2-Associated Coagulopathies
Source: Life (Basel). 2024 Feb 8;14(2):237. doi: 10.3390/life14020237 (PMC10890393; doi:10.3390/life14020237)
Supplement: Supplementary file 1 [file life-14-00237-s001.zip › life-2851565-supplementary.pdf]

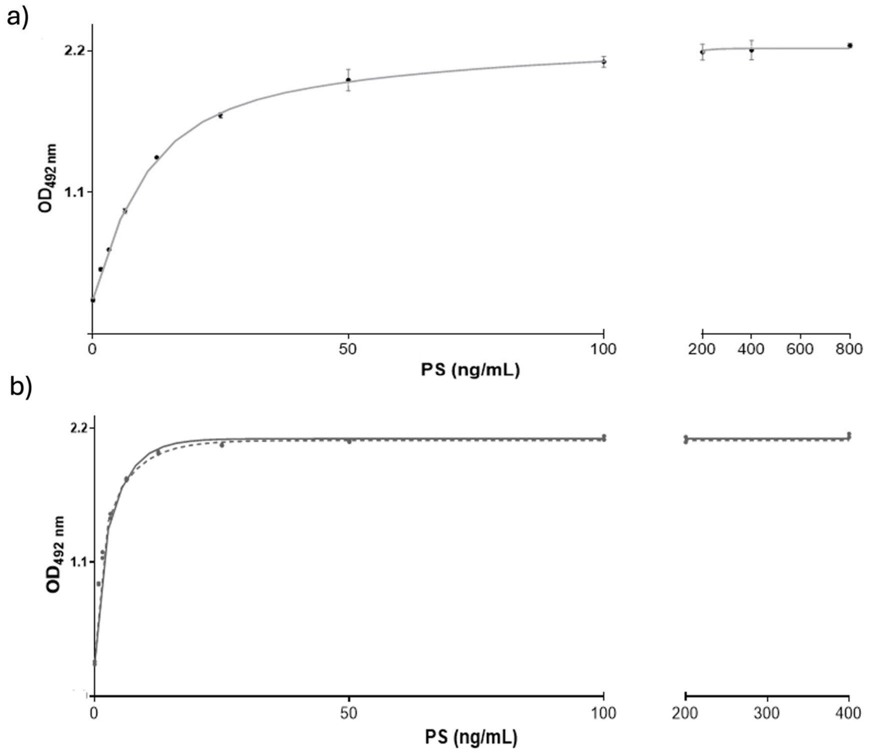

Figure S1. PROS1 ELISA sensitivity and specifity profile. (a) Increasing dilutions of pooled normal plasma (1/31.25—1/16,000, from 800 ng/mL to 1.56 ng/mL of PROS1) in PROS1 deficient plasma, were evaluated by ELISA sandwich, based on polyclonal antibodies. The very high reproducibility intra-assay is visible in the figure and the inter-assay variability is only 1.69%. Each point is the average of 3 measurements; (b) ELISA-PROS1 assay was conducted in pooled normal plasma by addition of C4BP protein (400 ng/mL, gray dashed line) or sample diluent (50 mM Tris, 150 mM NaCl, 5 mM CaCl2, pH 7.4, gray line) and no effect of this addition of this plasmatic physiological interactor of PROS1, was evaluable. This ELISA recognizes free and C4BP-bound PROS1 with identical affinity. Each point is the average of 2 evaluations.
